# Evaluation of the Effect of Hydroxyzine on Preoperative Anxiety and Anesthetic Adequacy in Children: Double Blind Randomized Clinical Trial

**DOI:** 10.1155/2021/7394042

**Published:** 2021-11-11

**Authors:** Esther Aleo, Amanda López Picado, Belén Joyanes Abancens, Carmen Soto Beauregard, Nuria Tur Salamanca, Carmen Esteban Polonios, María José Torrejón, Carlos González Perrino, Ana Rivas, Eva Arias, Diamelis Rodríguez, Mª. Ángeles Rivas, Marina Laura Rodríguez Rojo, Patricia Fernández García, Jaime Rodríguez Alarcón, Borja San Pedro de Urquiza

**Affiliations:** ^1^Unidad de Cuidados Intensivos Pediátricos y Unidad de Recuperación Postanestésica, Servicio de Pediatría, Instituto del Niño y del Adolescente, Hospital Clínico San Carlos, Instituto de Investigación Sanitaria del Hospital Clínico San Carlos (IdISSC), Madrid, Spain; ^2^Departamento de Pediatría, Facultad de Medicina, Universidad Complutense de Madrid, Madrid, Spain; ^3^Unidad de Investigación Clínica y Ensayos Clínicos, Hospital Clínico San Carlos, Instituto de Investigación Sanitaria del Hospital Clínico San Carlos (IdISSC), Madrid, Spain; ^4^Facultad de Salud, Universidad Internacional de la Rioja, Logroño, Spain; ^5^Servicio de Cirugía Pediátrica, Instituto del Niño y del Adolescente, Hospital Clínico San Carlos, Instituto de Investigación Sanitaria del Hospital Clínico San Carlos (IdISSC), Madrid, Spain; ^6^Coordinadora de la Unidad de Niños y Adolescentes del Instituto de Psiquiatría y Salud Mental, Instituto de Investigación Sanitaria del Hospital Clínico San Carlos (IdISSC), Madrid, Spain; ^7^Supervisora de Enfermería del Servicio de Pediatría, Instituto del Niño y del Adolescente, Hospital Clínico San Carlos, Instituto de Investigación Sanitaria del Hospital Clínico San Carlos (IdISSC), Madrid, Spain; ^8^Servicio de Análisis Clínicos, Hospital Clínico San Carlos, Instituto de Investigación Sanitaria del Hospital Clínico San Carlos (IdISSC), Madrid, Spain; ^9^Servicio de Anestesiología y Reanimación, Hospital Clínico San Carlos, Instituto de Investigación Sanitaria del Hospital Clínico San Carlos (IdISSC), Madrid, Spain; ^10^Departamento de Enfermería, Facultad Enfermería, Fisioterapia y Podología, Universidad Complutense de Madrid, Madrid, Spain; ^11^Departamento de Pediatría, Facultad de Medicina Universidad Francisco de Vitoria, Madrid, Spain

## Abstract

Surgical procedures can generate significant preoperative anxiety (POA) in as much as 70% of the paediatric population. The role of hydroxyzine and distractive techniques such as clowns in the management of anxiety is controversial. Our main objective was to evaluate the effect of hydroxyzine on the control of POA. The secondary objective was to assess the potential additive effect of hydroxyzine and distracting techniques. We performed a randomized double-blind, controlled clinical trial in children aged 2–16 years undergoing outpatient surgery (*n* = 165). Subjects were randomized to hydroxyzine (group 1) or placebo (group 2). For the secondary objective, two further groups were made by allocation by chance to hydroxyzine plus accompaniment with clowns (group 3) and placebo plus clowns (group 4). All patients were accompanied by their parents as the standard procedure. POA was determined by a modified Yale scale of POA (m-YPAS). Compliance of children during induction of anesthesia (Induction Compliance Checklist (ICC)) was also assessed. No differences (*p* = 0.788) were found in POA control at the time of induction measured by m-YPAS (group 1: 39.2 ± 27.9; group 2: 37.0 ± 26.1; group 3: 34.7 ± 25.5; group 4: 32.4 ± 20.5). No differences were found in the level of ICC between the different treatment arms (group 1: 1.8 ± 3.4; group 2: 1.5 ± 3.0; group 3: 1.2 ± 2.4; group 4: 1.5 ± 2.7). The combination of all treatments (group 3) was the only effective strategy to contain the progression of anxiety. In conclusion, hydroxyzine was not effective to control POA in children. The combination of hydroxyzine and clowns avoided the progression of POA in our patients. This trial is registered with ClinicalTrials.gov identifier: NCT03324828 (registered 21 September 2017, subject recruitment started on 12th January 2018).

## 1. Introduction

Anxiety in children triggered by a scheduled surgical intervention is a major issue due to its frequency, in as much as 70% of the paediatric population, with short-, medium-, and long-term consequences [1–5].

The need to have specific programs to reduce the anxiety of children is of special interest if we consider the adverse effects of surgery associated with high preoperative anxiety (POA) [3–8]. High levels of POA are responsible for increased surgical morbidity [3], postoperative analgesia needs [4], and increased number of days of hospitalization and rate of complications [1].

The management of POA in paediatric patients is a field under constant review, with the studies published to date having differed, controversial, and nonconclusive results [6–8].

To reduce POA, strategic programs that try to minimize the emotional impact have been designed. Some of these strategies are parental accompaniment during induction of anesthesia [6, 7], sedative premedication [8], and distraction techniques [9–12], including the presence of clowns [13] or music therapy [14].

Pharmacological studies performed with preoperative anxiolytic medication assess off-label drugs (such as clonidine) or drugs that require close monitoring and control measures (as midazolam) due to associated serious adverse events like delirium and respiratory depression [15, 16]. Hydroxyzine is an antihistamine with sedative properties approved for anxiolytic use both in Europe and the USA. Despite its widespread use in clinical practice, only few studies have assessed hydroxyzine's effectiveness, most of which have been in the context of minor odontology interventions [17, 18].

Furthermore, there are no clinical trials about its use for the management of POA in major outpatient paediatric surgery. To date, few studies have been conducted comparing the effectiveness of distracting anxiolytic techniques with the use of sedative drugs. And as far as we know, none of them have been oriented to study the possible additive effect of a combination of these interventions.

Therefore, the main objective of this study was to evaluate the efficacy of hydroxyzine for the control of POA and adequacy of anesthesia induction in major outpatient paediatric surgery.

## 2. Material and Methods

This study was carried out from 12th January 2018 till December 2020 in the Hospital Clinico San Carlos (HCSC), Madrid, Spain. The trial was registered prior to patient enrolment at ClinicalTrials.gov identifier: NCT03324828 (date of registration: October 30, 2017). This is a unicentric, randomized, controlled clinical trial with parallel groups randomized to a double blinded pharmacological intervention. Additional subgroups were made by nonrandomized assignment to distractive techniques. The trial design was published in TRIALS Aleo Luján et al. Trials (2020) 21 : 1 (10.1186/s13063-019-3906-2) [19].

### 2.1. Participant Recruitment

#### 2.1.1. Inclusion Criteria

The inclusion criteria were the following: (1) children aged between 2 and 16 years old, (2) American Society of Anesthesiologists physical status classification grades I and II, (3) informed consent (IC) signed by parents or legal guardians of the minors, and (4) specific informed consent for children aged between 12 and 16 years old.

#### 2.1.2. Exclusion Criteria

The exclusion criteria were the following: (1) patients who had undergone previous surgery at age 2 years or older; when children undergo operations at an age younger than 2 years old, they do not remember the surgical experience, and therefore, the variables analyzed are not influenced by this previous experience; (2) patients with confirmed allergy or hypersensitivity to the active substance, to any of the excipients, to cetirizine, to other derivatives of piperazine, to aminophylline, or to ethylenimine; (3) patients with porphyria; (4) patients with diagnosed prolongation of the QT interval (hydroxyzine is contraindicated); (5) patients with risk factors for QT interval prolongation, including preexisting cardiovascular disease, electrolyte balance disturbances (hypokalemia, hypomagnesaemia), family history of sudden cardiac death, significant bradycardia, and concomitant use of drugs with the potential to produce prolongation of the QT interval and/or induce Torsade de Pointes; and (6) allergy to antihistamines.

### 2.2. Grouping and Randomization of Participants

#### 2.2.1. Randomization

Following signature of the IC, randomization to treatment with hydroxyzine or placebo was performed. Randomization occurred in a 1 : 1 ratio in blocks of 8. Randomization was performed using REDCap. The sequence was blinded to all team members. In order to explore the potential additive effect of distracting anxiolytic techniques, two further groups with and without accompaniment by clowns (Dr. Sonrisas from Fundación Theodora) were made. Randomization to these latter groups was not possible due to the lack of availability of clowns every day. Thus, a nonrandomized group assignment depending on the clown presence/availability on the day of the intervention was done. Patients with and without clown accompaniment were assigned an alphabetical code.

#### 2.2.2. Intervention

The study participants were allocated to one of these strategies:

- *Group 1*. Pharmacological intervention (oral hydroxyzine 2 mg/kg masked with 5 ml of juice, administered at least 30 min prior to surgery) plus standard management consisting of parental accompaniment during the preoperative period, postanesthesia recovery area, and up to hospital discharge
- *Group 2*. Placebo (5 ml of juice) plus standard management
- *Group 3*. Hydroxyzine (as described in the previous groups) plus standard management combined with accompaniment and distraction by Dr. Sonrisas
- *Group 4*. Placebo plus standard management combined with distraction and accompaniment by Dr. Sonrisas during the preoperative period, postanesthesia recovery area, and up to hospital discharge

All the patients were video recorded to later evaluate the patient's state of anxiety by the modified Yale scale of preoperative anxiety m-YPAS scale. The m-YPAS scale considers that scores < 30 do not show anxiety.

#### 2.2.3. Blinding

To hide clown accompaniment to the evaluator of the m-YPAS, their appearance on the screen was avoided, and the recording was muted. The evaluation of all recordings was done by the principal investigator. Medication double-blinding was achieved by the administration of 5 ml of juice by a nonblinded nurse to all subjects, mixed or not with hydroxyzine, depending on group assignment.

### 2.3. Treatment Guideline

POA evaluation of all subjects in the study was performed at the following time-points:

- *Time-Point 0 (M0)*. Considered the baseline status because it is the moment when the patient arrives to the presurgical hospitalization area prior to being in contact with any method to reduce POA. This time-point was video recorded to evaluate the patient's baseline state of anxiety by the m-YPAS
- *Time-Point 1 (M1)*. During the stay in the presurgical hospitalization area, at least 30 min after receiving the assigned strategy. This time-point was video recorded to evaluate the patient's baseline state of anxiety by the m-YPAS
- *Time-Point 2 (M2)*. Moment when the patient is transferred to the operating room up to the entrance to the surgical ward and parental separation. The investigators recorded a film during the transfer to the operating room and up to the entrance to the surgical block to later evaluate a subject's preoperative anxiety by the m-YPAS scale
- *Time-Point 3 (M3)*. During the induction of anesthesia in the operating room, the induction of anesthesia was video recorded to later evaluate subject's preoperative anxiety by the m-YPAS scale. At this time-point, the anesthesiologist completed the Induction Compliance Checklist

### 2.4. Statistical Analysis and Sample Size

Anxiety in children was evaluated through comparing m-YPAS score between the moment of induction of anesthesia and the presurgical moment prior to the entrance to the operating room (M3). Sample size was calculated to allow detection of a difference of means of 14.3 points at m-YPAS between the group with parental accompaniment and hydroxyzine (mean: 18; standard deviation (SD): 13.5 points) compared to the group with parental accompaniment and placebo (mean: 32.3; SD: 24.2 points) [4, 20]. A sample size of 47 subjects in each group would have a power of 90%, with a level of significance of 0.025, to detect these differences. The final sample size calculation was 188 subjects (47 in each group).

### 2.5. Data Analysis

The analysis was performed by protocol and intention to treat. Qualitative varibles were summarized with the percentages and frequency. The quantitative variables were summarized with the mean and standard deviation (SD). The quantitative variables that showed an asymmetric distribution were summarized with the median and interquartile range (IQR). In the analysis of the association between qualitative variables, the chi-square test *χ*2 or Fisher's exact test was used, in the case that more than 25% of those expected were less than 5. For the comparison between quantitative and qualitative variables, the means were compared using the Student's *t*-test or the analysis of variance (ANOVA) or the Mann–Whitney *U* test or the Kruskal-Wallis test in case the quantitative variables were not adjusted to a normal distribution. In the case of analysis of ANOVA, the Tukey test was used. A significance value of 5% was accepted for all tests. The data processing and analysis was performed using the statistical package IBM Statistics SPSS 23.0.

#### 2.5.1. Safety

Adverse events were evaluated throughout the study. All adverse events were collected in the case report form for each subject, regardless of the causal relationship with study treatment. But no adverse event was registered.

### 2.6. Ethical Considerations

The study was approved by the Clinical Research Ethics Committee of the hospital. The confidentiality of subject data was always maintained in accordance with current legislation. Written informed consent was obtained before any intervention from all subjects, legal surrogates, parents, or legal guardians for minor subjects. This study was carried out following international ethical recommendations for conducting human research and clinical trials contained in the latest revision of the Declaration of Helsinki as well as those established in the Good Clinical Practice Guidelines and current legislation. All subjects were supervised by qualified medical personnel during their participation in the study.

## 3. Results

**Figure 1:**
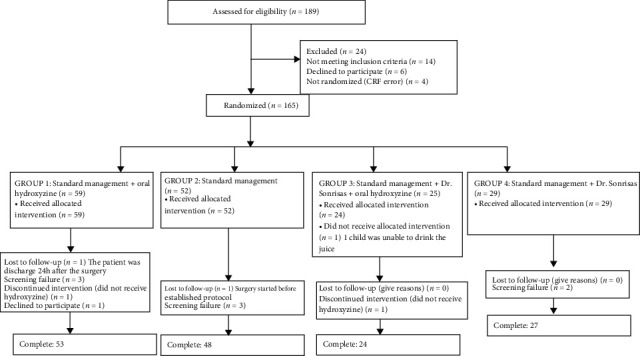
Flow chart.

We studied 165 patients with ages between 2 and 16 years (mean 7.4, SD 4.2). 127 were boys, and 41 were girls. The declaration of the COVID-19 global pandemic forced the premature ending of the study, and thus, the objective sample size was only reached in groups 1 (hydroxyzine) and 2 (placebo). Group distribution is summarized in Figure 1 (CONSORT flow, Graph 1: CONSORT flow chart). A total of 165 outpatient major surgery interventions were analyzed, distributed in phimosis surgeries (43%), cryptorchidism and/or inguinal hernia (11%), abdominal hernia (9%), skin cysts (7%), multiple surgeries (2%), and others (28%) (Table 1).

Anxiety was evaluated using the m-YPAS scale at the time of anesthetic induction (M3) in each of the therapeutic management groups. In group 1 (hydroxyzine + parents), a mean score of 38.4 (±27.5) was obtained, in group 2 (placebo + parents) 37.0 (±26.1), in group 3 (hydroxyzine + parents + clowns) 34.7 (±25.5), and in group 4 (placebo + parents + clowns) of 32.4 (±20.5). The mean scores of each treatment group were compared with each other at the time of induction, and no statically significant differences were found (*p* = 0.788) (Table 2).

**Figure 2:**
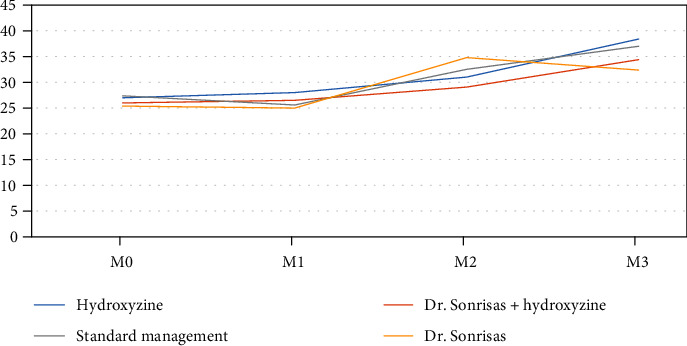
Anxiety levels in each moment and group.

Anxiety measured by m-YPAS obtained a maximum score in all groups at the time of induction (M3) except for group 4 (placebo + parents + clowns), which obtained the maximum anxiety score at M2 (Figure 2).

The adequacy of anesthetic induction evaluated using the ICC scale in the four treatment groups is reflected in Table 3. Overall, statistically significant differences were not detected between the different groups (*p* = 0.828) (Table 3).

We evaluated the evolution of anxiety with the m-YPAS scale throughout the whole surgical circuit in each of the four moments as referred to above.

At baseline (M0), the scores obtained were <30 in each of the branches, and no statistically significant differences were found (*p* = 0.624). However, when analyzing the progression of anxiety throughout the surgical circuit (from M0 to M3) in each of the branches, differences were observed according to the anxiolysis measures adopted.

- *Group 1* (Table 4). Significant differences were found between the different moments except between M0 and M1 (*p* = 0.861). Differences were observed in successive moments, with increasing anxiety as the moment of anaesthetic induction approached: between M0 27.7 (±10.3) and M2 31.9 (±12.1) (*p* = 0.013), between M0 27.7 (±10.3) and M3 38.4 (±27.7) (*p* = 0.010), between M1 28.0 (±12.2) and M2 31.9 (±12.1) (*p* = 0.025), and between M2 31.9 (±12.1) and M3 38.4 (±27.7) (*p* = 0.043)
- *Group 2* (Table 5). The evaluation of anxiety was not linearly ascending. First, a decrease was observed between M0 27.4 (±6.7) and M1 25.6 (±5.8) (*p* = 0.04). Subsequently, a significant increase in anxiety was observed between M0 and M2 32.5 (± 19.0) (*p* = 0.046) and between M0 and M3 37.0 (±26.1) (*p* = 0.010). Likewise, an increase in anxiety was found between M1 and M2 (*p* = 0.007) and between M1 and M3 (*p* = 0.002). However, there were no significant differences between M2 and M3 (*p* = 0.109)
- *Group 3* (Table 6). In this treatment group, unlike the rest of the groups analyzed, anxiety did not grow despite progressing in the circuit and approaching the moment of induction, with no significant differences in the m-YPAS scale between the different moments
- G*roup 4* (Table 7). There were differences between M0 25.4 (± 3.5) and M2 34.8 (± 21.7) (*p* = 0.03) and between M1 25.0 (± 4.1) and M2 34.8 (± 21.7) (*p* = 0.027), but no differences were found between the rest of the moments. As in group 1, there were no significant differences between M2 and M3 (*p* = 0.573)

Regarding safety, there were no adverse events in any group.

## 4. Discussion

The main objective of this study was to evaluate the efficacy of hydroxyzine vs. placebo in addition to the standard procedure of accompaniment by parents, for the control of POA. Our results show that this pharmacological intervention alone does not modify POA. However, our secondary objective was to assess the effect on POA of the combination of this pharmacological intervention with distractive techniques, and this strategy showed to be effective to contain the progression of anxiety in this paediatric population. The search for an ideal premedication drug to reduce POA in children is ongoing. The drugs used as anxiolytics are not exempt of side effects, and the studies about this issue are limited to dental procedures [17, 18, 21]. The role of clowns in the management of anxiety is controversial as there are studies both for and against them [13, 22–24]. We have not found any study that determines the additive effect on anxiolysis of distracting techniques with clowns and pharmacological treatment, as we propose here.

Studies that evaluate POA are difficult to perform and interpret, mostly because of the difficulty in assessing anxiety attributable to the surgical act. That is the reason why in the present study, POA was evaluated in different time-points. Most studies on this topic use a presurgical anxiety rating scale, the m-YPAS [20, 25–27]. According to some studies [4, 20, 25], the moment with the maximum rate of anxiety and fear associated with the entire surgical procedure is during anesthetic induction. Therefore, evaluating the child's anxiety during anesthetic induction is very useful to determine whether the strategies used in the presurgical period have been effective in reducing anxiety [20].

The m-YPAS scale considers 30 as the cut-off value, with scores lower than 30 for nonanxious children and 30 or higher to define anxiety. Taking these values into account, we can say that in our study, patients in all branches had a baseline situation (M0) and at the start of the surgical circuit (M1) without detectable anxiety by m-YPAS. However, when progressing and entering the surgical area (M2), all branches, except group 4 (which included the combination of all treatments), showed anxiety.

This can be explained by two fundamental reasons: in our institution, the accompaniment of both parents and the clown ends right at this point (M2); thus, the increase in anxiety in the groups could be explained by a cessation of the possible anxiolytic effect that both exerted until that moment. These results suggest that parental accompaniment until induction of anesthesia could reduce anxiety.

Humour and laughter have characteristics that could help reduce pain and stress, but the information available is controversial; they seem to reduce anxiety in hospitalization prior to the operation room, but it has not yet been possible to demonstrate their benefit as anxiolytic therapy within the surgical area [22]. In a subsequent study, Vagnoli et al. concluded that the combination of clowns and parental accompaniment during the preoperative stage achieved a higher reduction of POA than either parental accompaniment alone or oral premedication with midazolam [24]. In our trial, clowns' therapy is interrupted at M2, observing an increase of anxiety at this time-point in group 2 but also in group 1 (probably for the same reason) and group 3. So, more studies with a different design are needed to confirm that.

On the other hand, unlike the rest of the branches, we did not find significant differences in the level of anxiety between the different time points in group 3. Thus, according to our results, the addition of anxiolytic methods, represented in this group, was the only strategy that prevented the progression of anxiety throughout the surgical circuit.

If we compare anxiety at the time of anesthetic induction between the 4 different branches of POA management, we did not find significant differences between them. Consequently, we could not speak of superiority of any intervention if we analyze a specific moment measured both with the m-YPAS as with the ICC.

Our study has some limitations that must be considered: As a result of the situation caused by the COVID-19 pandemic, the study had to be closed prematurely. The cessation of the planned surgical activity and the prohibition of visits by clowns to the hospital prevented the study from continuing during the pandemic. The initially estimated sample size was 188 patients, and due to this premature closure, only 165 data could be collected. Target sample size was not reached in groups 3 and 4, so results for these groups must be considered with caution. However, sample size was above the target for groups 1 and 2, so the results for our primary objective are statistically valid.

The clowns were not always at the hospital; therefore, assignment to this intervention could not be randomized and depended on whether they were present or not on the day of surgery. To reduce the risk of bias, the healthcare professionals that planned the patient's surgery did not know when the clowns would be present. However, at the close of this study, we found that the distribution of the 4 designated groups was not homogeneous, with less patients in those groups with accompaniment by clowns (groups 3 and 4). Despite this, we did not find differences between the 4 groups in the m-YPAS analysis at any time or in the ICC.

The predominance of males in the cohort reflects the high prevalence of phimosis surgery in the study population, possibly reflecting the real-world population of paediatric MAS. The use of video recording, and in particular, the presence of a person operating the camera could have modified the patient's normal behaviour, as proposed previously. However, interaction between the cameraman and the child was avoided in our study to minimize this risk.

Another limitation of our study is the wide age range, from 2 to 16 years. It is possible that the same strategy may not work for all ages.

## 5. Conclusions

Anxiety in children increases progressively along the preoperative circuit. The use of hydroxyzine vs. placebo is not useful to reduce POA. However, the combination of anxiolytic premedication with hydroxyzine together with the presence of the parents and distracting therapy with clowns demonstrated to be the most effective strategy to prevent the progression of POA in our study. We found that anxiety increased significantly when clown accompaniment and distraction were interrupted before entering the surgical area. Thus, distracting techniques should be maintained in these patients until the moment of anaesthetic induction. Further studies are needed to determine the optimal management of POA in the paediatric population.

## Figures and Tables

**Table 1:** Demographic characteristics of the patients.

		Standard management + oral hydroxyzine (*n* = 59)	Standard management (*n* = 52)	Standard management + Dr. Sonrisas + oral hydroxyzine (*n* = 25)	Standard management + Dr. Sonrisas (*n* = 29)	Total
Age (years)	Mean ± SD^∗^	7.7 ± 4.1	8.0 ± 4.3	7.7 ± 4.1	6.4 ± 3.3	7.4 ± 4.2
Sex (*n*, %)	Male	19 (76.0)	40 (74.1)	19 (76.0)	21 (72.4)	127 (74.6)
	Female	6 (24.0)	12 (22.2)	6 (24.0)	8 (27.6)	41 (25.4)
Type of surgery						
	Phimosis	9 (37.5)	27 (52.9)	9 (37.5)	14 (48.3)	71 (43)
	Cryptorchidism and/or hernia	2 (8.3)	4 (7.8)	2 (8.3)	4 (13.8)	18 (10.9)
	Abdominal hernia	1 (4.2)	3 (5.9)	1 (4.2)	1 (3.4)	15 (9.5)
	Cutaneous cyst	2 (8.3)	2 (3.9)	2 (8.3)	1 (3.4)	11 (6.7)
	Multiple surgeries	0 (0)	0 (0)	0 (0)	1 (3.4)	4 (2.4)
	Other	10 (41.7)	15 (29.4)	10 (41.7)	8 (27.6)	46 (27.9)

^∗^SD: standard deviation.

**Table 2:** m-YPAS at the time of anaesthetic induction.

	Mean ± SD^∗^	*p*
Standard management + oral hydroxyzine (*n* = 59)	39.2 ± 27.9	0.788
Standard management (*n* = 52)	37.0 ± 26.1	
Standard management + Dr. Sonrisas + oral hydroxyzine (*n* = 25)	34.7 ± 25.5	
Standard management + Dr. Sonrisas (*n* = 29)	32.4 ± 20.5	
Total	36.6 ± 25.7	

^∗^SD: standard deviation.

**Table 3:** ICC values in each group.

	Mean ± SD^∗^	Standard management + oral hydroxyzine (*n* = 59)	Standard management (*n* = 52)	Standard management + Dr. Sonrisas + oral hydroxyzine (*n* = 25)	Standard management + Dr. Sonrisas (*n* = 29)
Standard management + oral hydroxyzine (*n* = 59)	1.8 ± 3.4		0.930	0.864	0.961
Standard management (*n* = 52)	1.5 ± 3.0			0.989	1.000
Standard management + Dr. Sonrisas + oral hydroxyzine (*n* = 25)	1.2 ± 2.4				0.993
Standard management + Dr. Sonrisas (*n* = 29)	1.5 ± 2.7				
Total	1.5 ± 3.0				

^∗^SD: standard deviation.

**Table 4:** Correlation values in standard management + oral hydroxyzine group.

m-YPAS	Mean ± SD^∗^	M0	M1	M2	M3
M0	27.7 ± 10.3				
M1	28.0 ± 12.2	0.861			
M2	31.9 ± 12.1	0.013	0.025		
M3	38.4 ± 27.7	0.010	0.006	0.043	

^∗^SD: standard deviation.

**Table 5:** Correlation values in standard management group.

m-YPAS	Mean ± SD^∗^	M0	M1	M2	M3
M0	27.4 ± 6.7				
M1	25.6 ± 5.8	0.04			
M2	32.5 ± 19.0	0.046	0.007		
M3	37.0 ± 26.1	0.01	0.002	0.109	

^∗^SD: standard deviation.

**Table 6:** Correlation values in standard management + Dr. Sonrisas + oral hydroxyzine group.

m-YPAS	Mean ± SD^∗^	M0	M1	M2	M3
M0	26.5 ± 6.2				
M1	26.4 ± 6.8	0.964			
M2	29.1 ± 10.7	0.150	0.094		
M3	34.7 ± 25.5	0.116	0.069	0.174	

^∗^SD: standard deviation.

**Table 7:** Correlation values in standard management + Dr. Sonrisas group.

m-YPAS	Mean ± SD^∗^	M0	M1	M2	M3
M0	25.4 ± 3.5				
M1	25.5 ± 4.1	0.588			
M2	34.8 ± 21.7	0.03	0.027		
M3	32.4 ± 20.5	0.092	0.082	0.573	

^∗^SD: standard deviation.

## Data Availability

Regarding sharing materials and managing of intellectual property, data will be available on request to the authors.
